# Increased participation and improved performance in age group backstroke master swimmers from 25–29 to 100–104 years at the FINA World Masters Championships from 1986 to 2014

**DOI:** 10.1186/s40064-016-2209-2

**Published:** 2016-05-17

**Authors:** Chiara M. Unterweger, Beat Knechtle, Pantelis T. Nikolaidis, Thomas Rosemann, Christoph A. Rüst

**Affiliations:** Institute of Primary Care, University of Zurich, Zurich, Switzerland; Facharzt FMH für Allgemeinmedizin, Gesundheitszentrum St. Gallen, Vadianstrasse 26, 9001 St. Gallen, Switzerland; Department of Physical and Cultural Education, Hellenica Army Academy, Athens, Greece

**Keywords:** Age group, Swimming, Master athlete, Performance, Sex differences

## Abstract

Participation and performance trends in age group athletes have been investigated for different sport disciplines, but not for master swimmers. The knowledge on this topic is still missing for a particular stroke such as backstroke. Changes in participation and performance of male and female age group backstroke swimmers (≥25 years) competing in 50, 100 and 200 m pool swimming at the FINA World Masters Championships held between 1986 and 2014 were investigated using mixed-effects regression analyses. The overall participation was *n* = 26,217 including *n* = 13,708 women and *n* = 12,509 men. In 50 m, female (age groups 85–89 years; *p* = 0.002) and male participation (age groups 55–59; *p* = 0.030 and 80–84 years; *p* = 0.002) increased, while female participation decreased in age groups 55–59 (*p* = 0.010) and 60–64 years (*p* = 0.050). In 100 and 200 m, participation increased in age groups 45–49, 50–54, 65–69, 70–74, 80–84 years. Swimmers in age groups 25–29 to 95–99 years improved performance over all distances. Women were slower than men in age groups 25–29 to 80–84 years, but not in age groups 85–89 to 95–99 years over all distances. In 50 m and 100 m, the sex difference decreased in age groups 40–44 (*p* = 0.007 and *p* = 0.005), 45–49 (*p* = 0.017 and *p* = 0.034), 50–54 (*p* = 0.002 and *p* = 0.040), to 55–59 years (*p* = 0.002 and *p* = 0.004). In 200 m, the sex difference decreased in age groups 40–44 (*p* = 0.044) and 90–94 (*p* = 0.011), but increased in age group 25–29 years (*p* = 0.006). In summary, in age group backstroke swimmers, (1) participation increased or remained unchanged (except women in age groups 55–59 and 60–64 years in 50 m), (2) swimming performance improved in all age groups from 25–29 to 95–99 years over all distances, (3) men were faster than women in age groups 25–29 to 80–84 years (except age groups 85–89 to 95–99 years) over time and all distances.

## Background

Master athletes are generally defined as athletes older than 35 years, training for and competing in organized forms of sport specifically designed for older athletes (www.world-masters-athletics.org/about-us). Due to the increasing life expectancy in the general Western population (Oeppen and Vaupel 2002; www.who.int/mediacentre/news/releases/2014/world-health-statistics-2014/en/), the knowledge of changes in participation and performance of master athletes in different sport disciplines is of high interest for physicians, coaches and athletes.

In recent years, there has been a considerable increase in participation of master athletes and in several age groups athletes also improved performance in different endurance sport disciplines such as marathon (Anthony et al. 2014; Jokl et al. 2004) and ultra-marathon running (Jampen et al. 2013; Rüst et al. 2013; Zingg et al. 2013) ultra-cycling (Shoak et al. 2013), long-distance triathlon such as Ironman triathlon (Lepers et al. 2013; Stiefel et al. 2013, 2014) and Triple and Deca Iron ultra-triathlon (Knechtle et al. 2012), long-distance inline-skating (Teutsch et al. 2013) and ultra-mountain biking (Haupt et al. 2013). Although large cross-sectional studies in swimming already investigated changes of age, performance (König et al. 2014) and sex differences (Wild et al. 2014) in World Championships, cross- sectional studies investigating participation and performance trends of master backstroke swimmers are missing.

Despite the abovementioned general definition according to which master athletes were defined as those older than 35 years (Reaburn and Dascombe 2008), this definition might vary by sport disciplines. In swimming, the term ʽmaster athleteʼ defines swimmers of 25 years in age or older following the Fédération Internationale de Natation-FINA (www.fina.org/H2O/). In swimming, several studies investigated swimming performance in different age groups from 25 to 80 years (Bongard et al. 2007; Rüst et al. 2012; Senefeld et al. 2016; Vaso et al. 2013; Wolfrum et al. 2013) and 40–79 years (Akkari et al. 2015). Other authors concentrated on the age-related performance decline, but focused primarily on freestyle (Bongard et al. 2007; Donato et al. 2003; Fairbrother 2007; Rubin et al. 2013; Tanaka and Seals 1997). In recent years, however, for other strokes such as butterfly (Senefeld et al. 2016; Zingg et al. 2014a, b), breaststroke (Koch-Ziegenbein et al. 2013; Senefeld et al. 2016; Wolfrum et al. 2013, 2014), individual medley (Buhl et al. 2013a, b; Vaso et al. 2013) and backstroke (Janoschka et al. 2014; Kollarz et al. 2013a, b; Senefeld et al. 2016) changes in the age of peak swimming performance were investigated.

Although the age of peak swimming performance is important to plan an athletic career for elite swimmers, the knowledge of sex differences in swimming performance is also of high interest. Several authors showed that the sex difference in swimming performance decreased with increasing race distance for freestyle (Rüst et al. 2012; Tanaka and Seals 1997), individual medley (Buhl et al. 2013a), breaststroke (Wolfrum et al. 2013, 2014) and butterfly (Zingg et al. 2014a, b). Most of these studies investigated rather shorter time periods of 5–11 years (Rüst et al. 2012; Tanaka and Seals 1997; Wolfrum et al. 2013; Zingg et al. 2014a). A recent study, covering a time period of 26 years in swimming, reported that in all 5-year age groups of top ten master swimmers aged between 25 and 89 years men were faster than women for freestyle, backstroke and breaststroke, with the greatest sex difference being observed in butterfly (Senefeld et al. 2016).

The knowledge of participation and performance trends in swimming in different age groups for a particular stroke such as backstroke is still missing. It has been reported that even master backstroke swimmers aged 100 years and older can set world records in their age groups (www.washingtonpost.com/blogs/early-lead/wp/2015/04/06/meet-the-100-year-old-japanese-swimmer-who-set-a-1500-meter-world-record/; http://swimswam.com/jaring-timmerman-oldest-masters-swimmer-passes-away-105/). A particularity of backstroke swimming is that the respiratory tracts are not under water, a fact that might be preferred especially by very old master swimmers.

To the best of our knowledge, there are no studies focusing on changes in participation and performance of world class level master swimmers in different age groups competing in backstroke swimming. This information would be important for swimmers and professionals involving in their training (e.g. coaches, fitness trainers), as well as for researchers focusing on the study of older athletes as a model of successful aging. The aim of the present study was to investigate participation and performance trends of master athletes older than 25 years in different age groups competing in backstroke pool swimming for distances including 50, 100 and 200 m at the FINA World Masters Championships across the years 1986–2014. Based upon recent reports, we hypothesized for male and female master backstroke swimmers that (1) participation would increase for all age groups over time and (2) swimming performances would improve for all age groups over time. Based on the recent findings of Senefeld et al. (2016), we hypothesized that (3) men would be faster than women in master backstroke swimming in all age groups across years.

## Methods

### Ethics

All procedures used in the study were approved by the Institutional Review Board of Kanton St. Gallen, Switzerland. A waiver of the requirement for informed consent of the participants was granted given the fact that the study involved the analysis of publicly available data.

### Data sampling and data analysis

All athletes (*n* = 26,217; 13,708 women and 12,509 men) competing in the FINA World Masters Championships between 1986 and 2014 in all age groups over courses from 50 to 200 m were analysed for trends in participation and performance over long and short courses. To avoid a selection bias, all backstroke swimmers ranked in an age group were considered and there was not further consideration of top swimmers in an age group or regarding results of qualifying, semi-final or final races. In the FINA World Masters Championships, all athletes who successfully complete a race are ranked in their age group with no selection. Master swimmers competing in the FINA World Masters Championships were defined as athletes equal or older than 25 years. All data were obtained from the official and publicly accessible FINA website (www.fina.org/H2O/). FINA records in the FINA World Masters Championships all competitors in 5-years age groups from 25–29 to 105–109 years. All races of backstroke swimming (50, 100, 200 m), which were held from 1986 to 2014, were included. From 1986 to 2014, the FINA World Masters Championships were held in Japan, Australia, Brazil, USA, Canada, Great Britain, Morocco, Germany, New Zealand, Italy and Sweden (www.fina.org/H2O/). Mean race time for all competitors in a specific age group was calculated for each year.

### Statistical analysis

Trends in participation were analysed using single linear regression models. A mixed-effects regression model with finisher as random variable to consider finishers who completed several races was used to investigate changes in performance. We included sex, distance and calendar year as fixed variables. We also considered interaction effects between sex and distance and the final model was selected by means of Akaike information Criterion (AIC). Sex difference was calculated using the equation 100 × ([time in women] − [time in men]/[time in men]). A two-way analysis of variance (ANOVA) examined the distance × calendar year interaction on race time. The effect size in the ANOVA was evaluated using (η^2^) as following: trivial (η^2^ < 0.01), small (0.01 ≤ η^2^ < 0.06), medium (0.06 ≤ η^2^ < 0.14) and large (η^2^ ≥ 0.14). The men-to-women ratio was calculated for each year and race distance when each sex group consisted by at least five participants. Statistical analyses were performed using IBM SPSS Statistics (Version 22, IBM SPSS, Chicago, IL, USA). Significance was accepted at *p* < 0.05 (two-sided for *t* tests). Data in the text and tables are presented as mean ± standard deviation (SD).

## Results

### Participation trends

**Fig. 1:**
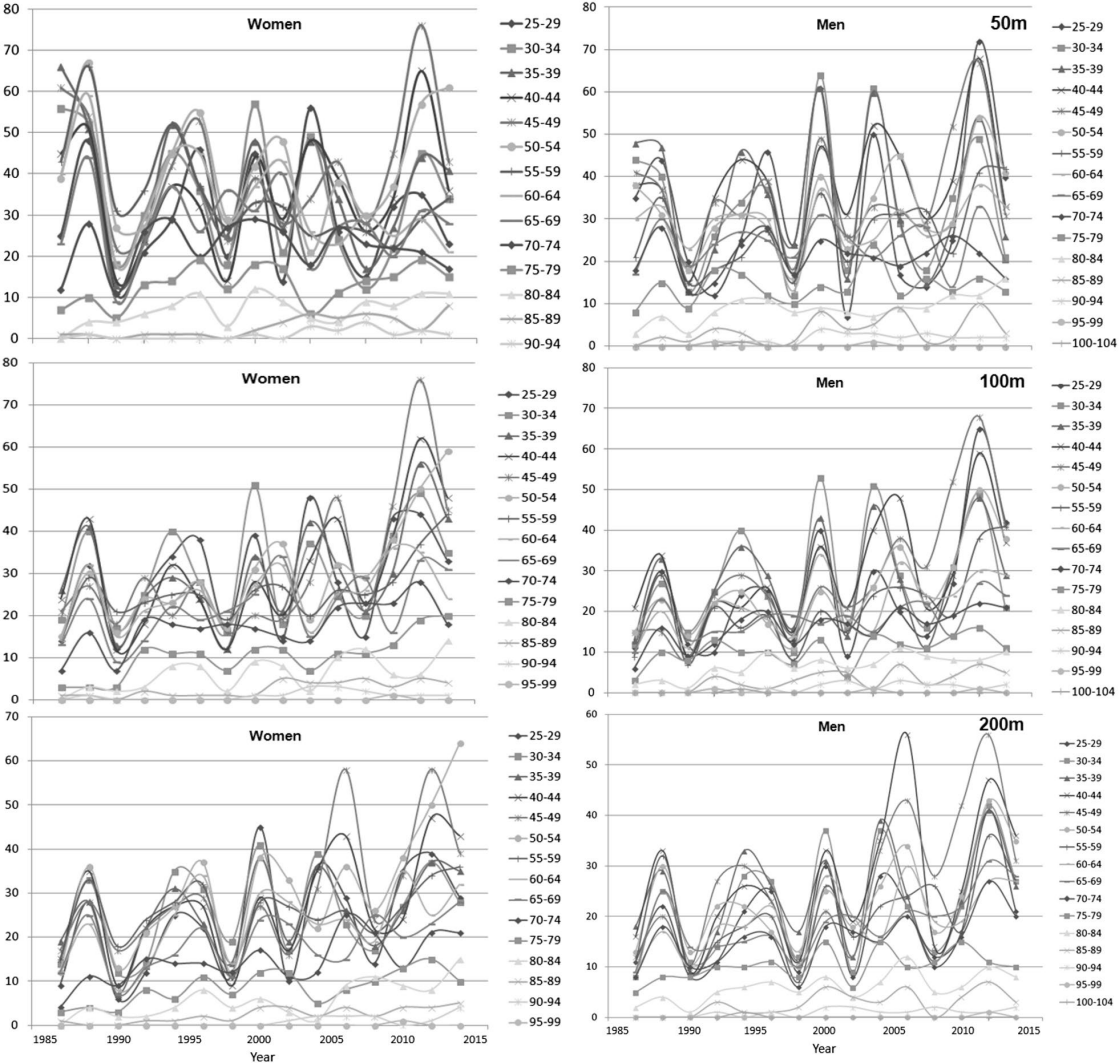
Participation of women and men across years over 50, 100 and 200 m

**Fig. 2:**
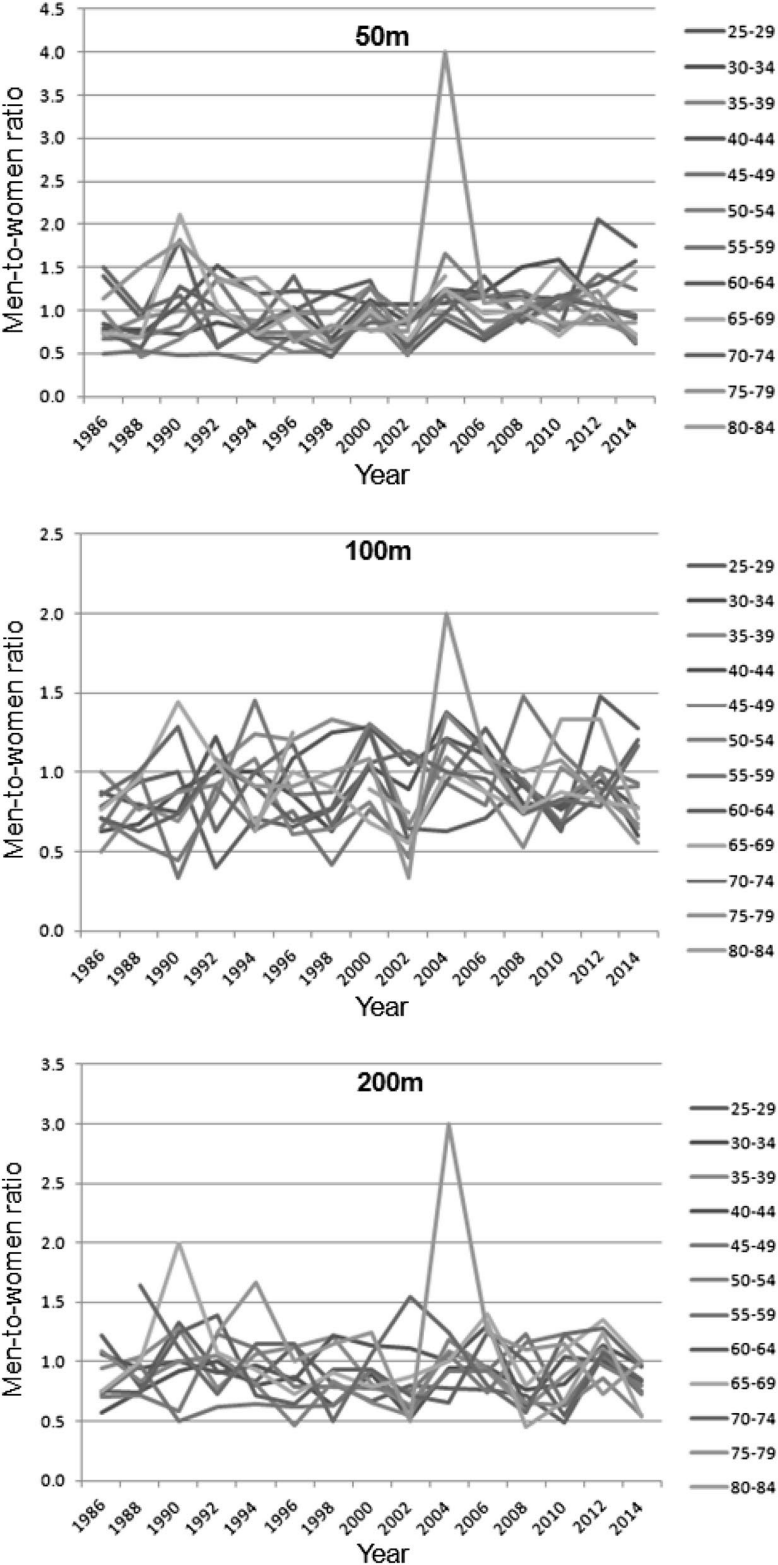
Men-to-women ratio across years over 50, 100 and 200 m distance

 Figure 1 presents the participation of women and men across years in different age groups from 50 to 200 m distance. In 50 m, female participation increased in age groups 85–89 (*p* = 0.002) years and male participation in age groups 55–59 (*p* = 0.030) and 80–84 (*p* = 0.002) years, while female participation decreased in age groups 55–59 (*p* = 0.010) and 60–64 (*p* = 0.050) years. In 100 m, female and male participation increased in age groups 40–44 (female: *p* = 0.031; male: *p* = 0.025), 45–49 (female: *p* = 0.010; male: *p* = 0.000), 50–54 (female: *p* = 0.001; male: *p* = 0.003), 55–59 (female: *p* = 0.006; male: *p* = 0.010), 60–64 (female: *p* = 0.068; male: *p* = 0.011), 65–69 (female: *p* = 0.019; male: *p* = 0.028), 70–74 (female: *p* = 0.003; male: *p* = 0.002), 75–79 (female: *p* = 0.000; male: *p* = 0.036), 80–84 (female: *p* = 0.026; male: *p* = 0.001) years. In 200 m, participation of both men and women increased in age groups 45–49 (female: *p* = 0.014; male: *p* = 0.005), 50–54 (female: *p* = 0.006; male: *p* = 0.027), 60–64 (female: *p* = 0.025; male: *p* = 0.008), 65–69 (female: *p* = 0.039; male: *p* = 0.006), 70–74 (female: *p* = 0.002; male: *p* = 0.024) and 80–84 (female: *p* = 0.004; male: *p* = 0.006) years. No consistent trend was observed in the men-to-women ratio across years (Fig. 2). We were unable to perform regression analysis in age groups 100–104 and 95–99 years (except for 50 m for men); since there were too few participants in these age groups.

### Performance trends

**Fig. 3:**
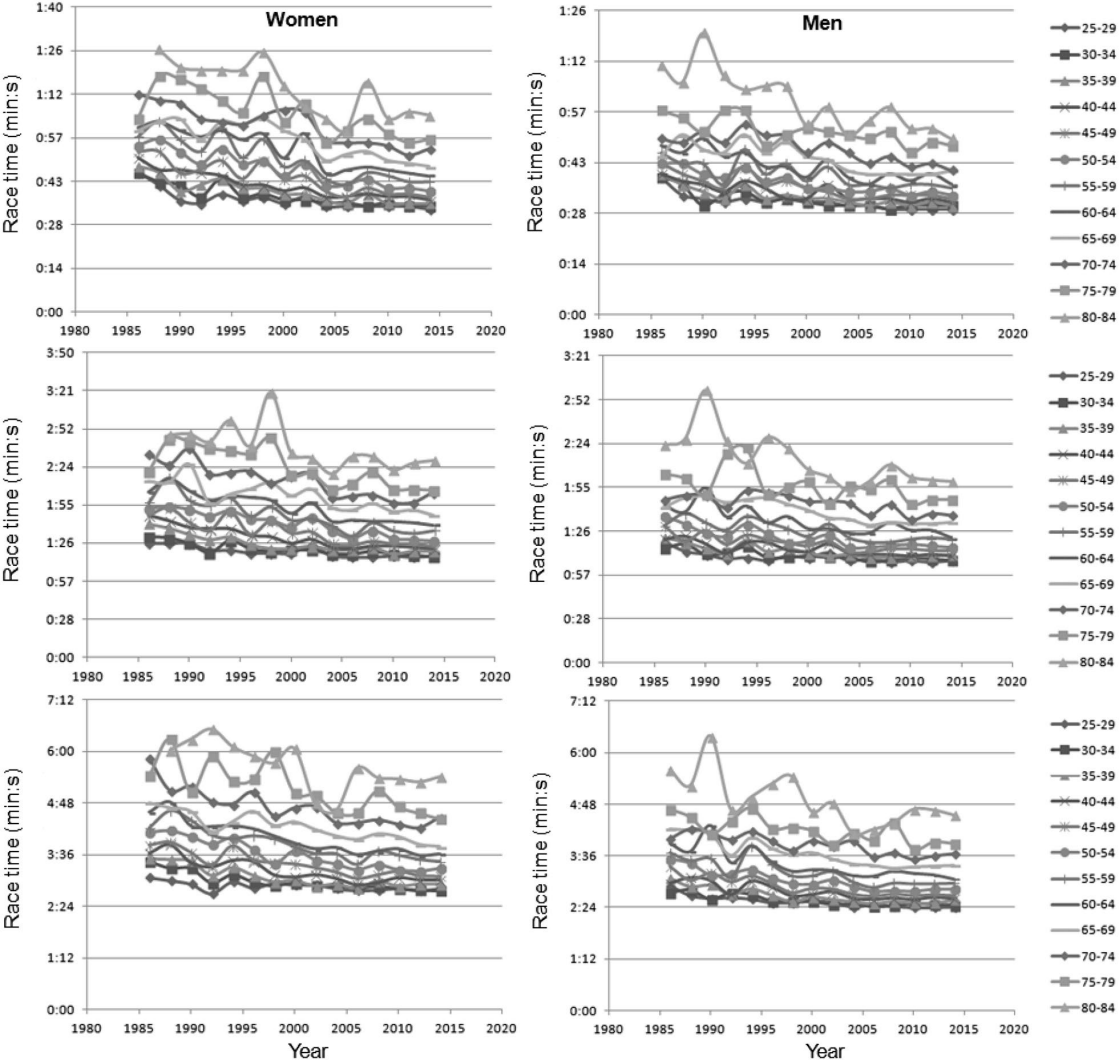
Race time (min:s) across years for women and men over 50, 100 and 200 m distance

Results of the mixed-effects regression analyses for performances in age groups are shown in Table 1. In age groups 25–29 to 95–99 years, male and female performance improved across years (Fig. 3). Over 50 m, race times for women in 2014 in age groups from 25–29 to 90–94 were as followed 00:34, 00:36, 00:37, 00:38, 00:40, 00:43, 00:45, 00:48, 00:54, 00:57, 01:05, 01:24, 01:17 min:s. In 2014 race times for women competing in 100 m backstroke swimming in age groups from 25–29 to 90–94 were 01:17, 01:16, 01:20, 01:22, 01:24, 01:28, 01:36, 01:40, 01:47, 02:05, 02:06, 02:29, 02:40, 02:39 min:s. Over 200 m distance race times for women in age groups from 25–29 to 90–94 in 2014 were 02:49, 02:46, 02:55, 03:01, 03:06, 03:17, 03:26, 03:37, 03:47, 04:28, 04:26, 05:25, 05:39, 06:20 min:s. Women were slower than men in age groups 25–29 to 80–84 years. There was a positive interaction between female sex and increasing race distance (*i.e.* from 50 m to 200 m). In age groups 85–89 (*p* = 0.064), 90–94 (*p* = 0.319) and 95–99 years (*p* = 0.053), no sex difference in performance was observed. In age groups 85–89 years, there was a positive interaction between female sex and 200 m, but not for 100 m (*p* = 0.097). In age groups 90–94 (*p* = 0.319) and 95–99 years (*p* = 0.053), no interaction between female sex and race distance could be found. A two-way ANOVA showed a distance × calendar year interaction on race time of all swimmers (*p* < 0.001, η^2^ = 0.014), women (*p* < 0.001, η^2^ = 0.017) and men (*p* < 0.001, η^2^ = 0.017), according to which there was a larger decrease of race time in 200 m than in the smaller distances. The magnitude (small) of this trend was similar in both sexes.

**Table 1:** Results of the mixed-effects regression analyses for performances in age groups

Parameter	Estimate	*p*	Parameter	Estimate	*p*	Parameter	Estimate	*p*
*25–29 years*			*50–54 years*			*75–79 years*		
Constant term	32.54	<0.0001	Constant term	38.70	<0.0001	Constant term	53.91	<0.0001
[sex = women]	5.75	<0.0001	[sex = women]	9.05	<0.0001	[sex = women]	14.61	<0.0001
[distance = 200 m]	119.55	<0.0001	[distance = 200 m]	142.56	<0.0001	[distance = 200 m]	200.59	<0.0001
[distance = 100 m]	37.11	<0.0001	[distance = 100 m]	44.86	<0.0001	[distance = 100 m]	66.44	<0.0001
[sex = women] × [distance = 200 m]	14.78	<0.0001	[sex = women] × [distance = 200 m]	25.30	<0.0001	[sex = women] × [distance = 200 m]	35.83	<0.0001
[sex = women] × [distance = 100 m]	5.19	<0.0001	[sex = women] × [distance = 100 m]	9.02	<0.0001	[sex = women] × [distance = 100 m]	8.40	0.003
*30–34 years*			*55–59 years*			*80–84 years*		
Constant term	33.51	<0.0001	Constant term	40.41	<0.0001	Constant term	57.63	<0.0001
[sex = women]	6.49	<0.0001	[sex = women]	12.89	<0.0001	[sex = women]	17.64	<0.0001
[distance = 200 m]	120.87	<0.0001	[distance = 200 m]	151.47	<0.0001	[distance = 200 m]	229.22	<0.0001
[distance = 100 m]	37.81	<0.0001	[distance = 100 m]	47.39	<0.0001	[distance = 100 m]	74.41	<0.0001
[sex = women] × [distance = 200 m]	18.36	<0.0001	[sex = women] × [distance = 200 m]	28.70	<0.0001	[sex = women] × [distance = 200 m]	34.66	<0.0001
[sex = women] × [distance = 100 m]	5.70	<0.0001	[sex = women] × [distance = 100 m]	9.48	<0.0001	[sex = women] × [distance = 100 m]	9.70	0.023
*35–39 years*			*60–64 years*			*85–89 years*		
Constant term	34.86	<0.0001	Constant term	42.95	<0.0001	Constant term	67.74	<0.0001
[sex = women]	6.32	<0.0001	[sex = women]	13.44	<0.0001	[sex = women]	15.90	0.064
[distance = 200 m]	125.69	<0.0001	[distance = 200 m]	160.22	<0.0001	[distance = 200 m]	246.22	<0.0001
[distance = 100 m]	39.35	<0.0001	[distance = 100 m]	50.53	<0.0001	[distance = 100 m]	79.94	<0.0001
[sex = women] × [distance = 200 m]	18.34	<0.0001	[sex = women] × [distance = 200 m]	25.20	<0.0001	[sex = women] × [distance = 200 m]	46.33	<0.0001
[sex = women] × [distance = 100 m]	6.54	<0.0001	[sex = women] × [distance = 100 m]	10.14	<0.0001	[sex = women] × [distance = 100 m]	14.57	0.097
*40–44 years*			*65–69 years*			*90–94 years*		
Constant term	35.50	<0.0001	Constant term	47.13	<0.0001	Constant term	83.35	<0.0001
[sex = women]	8.07	<0.0001	[sex = women]	12.57	<0.0001	[sex = women]	16.92	0.319
[distance = 200 m]	129.97	<0.0001	[distance = 200 m]	172.40	<0.0001	[distance = 200 m]	300.85	<0.0001
[distance = 100 m]	40.47	<0.0001	[distance = 100 m]	54.57	<0.0001	[distance = 100 m]	93.95	<0.0001
[sex = women] × [distance = 200 m]	20.37	<0.0001	[sex = women] × [distance = 200 m]	25.30	<0.0001	[sex = women] × [distance = 200 m]	26.36	0.192
[sex = women] × [distance = 100 m]	7.15	<0.0001	[sex = women] × [distance = 100 m]	9.99	<0.0001	[sex = women] × [distance = 100 m]	13.51	0.457
*45–49 years*			*70–74 years*			*95–99 years*		
Constant term	36.47	<0.0001	Constant term	49.49	<0.0001	Constant term	109.78	<0.0001
[sex = women]	9.76	<0.0001	[sex = women]	14.96	<0.0001	[sex = women]	−62.41	0.053
[distance = 200 m]	135.16	<0.0001	[distance = 200 m]	182.98	<0.0001	[distance = 200 m]	379.98	<0.0001
[distance = 100 m]	42.44	<0.0001	[distance = 100 m]	57.90	<0.0001	[distance = 100 m]	134.31	<0.0001
[sex = women] × [distance = 200 m]	20.64	<0.0001	[sex = women] × [distance = 200 m]	32.21	<0.0001	[sex = women] × [distance = 200 m]	16.57	0.499
[sex = women] × [distance = 100 m]	6.50	<0.0001	[sex = women] × [distance = 100 m]	11.00	<0.0001	[sex = women] × [distance = 100 m]

### Changes in sex difference in performance

**Fig. 4:**
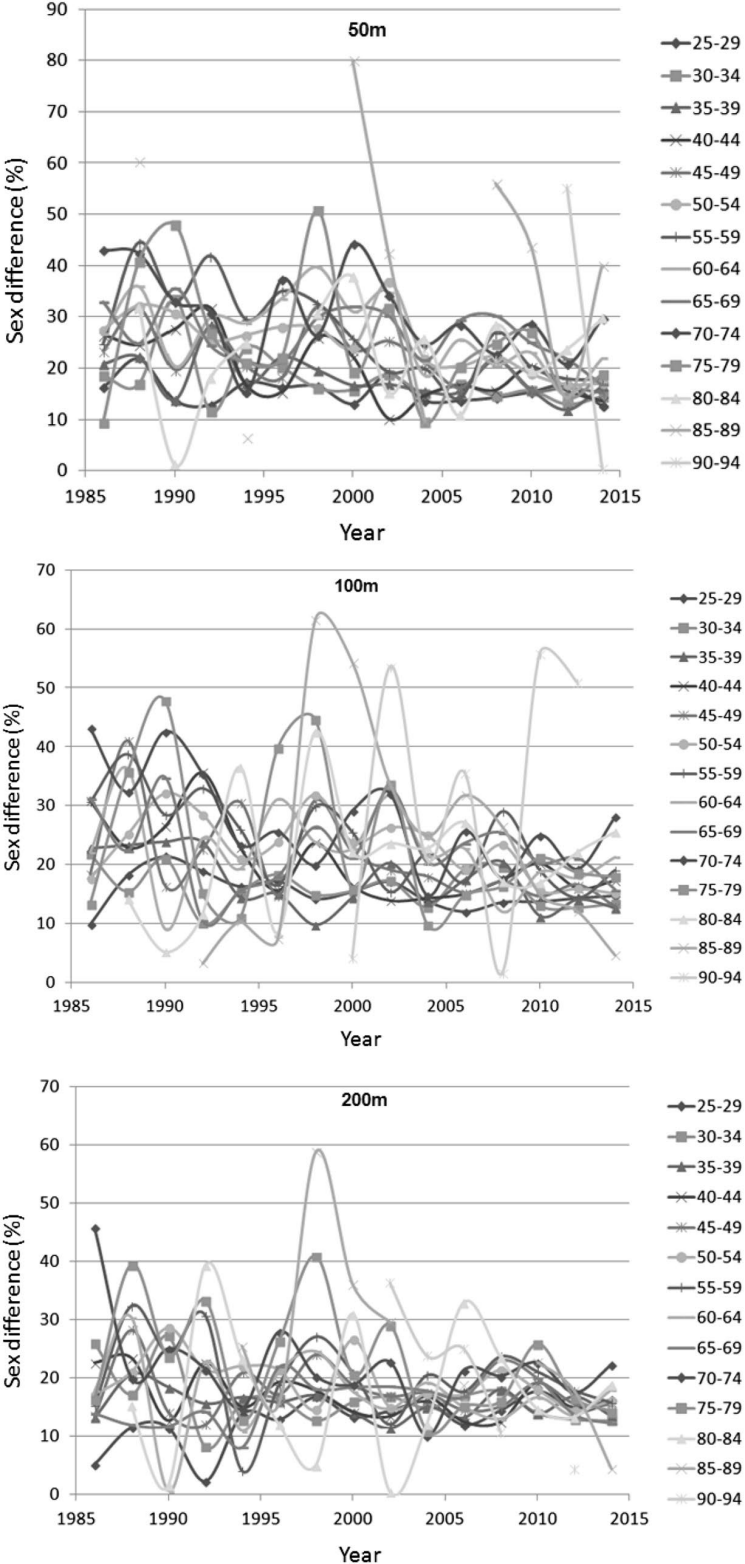
Sex differences (%) across years over 50, 100 and 200 m distance

Sex differences from 50 to 200 m distance across years are shown in Fig. 4. Regression analysis could be performed for age groups 25–29 to 90–94 years. In 50 m, the sex difference decreased significantly in age groups 40–44 (*p* = 0.007), 45–49 (*p* = 0.017), 50–54 (*p* = 0.002) and 55–59 (*p* = 0.002) years. In 100 m, a significant decrease in sex difference for age groups 35–39 (*p* = 0.015), 40–44 (*p* = 0.005), 45–49 (*p* = 0.034), 50–54 (*p* = 0.040), 55–59 (*p* = 0.004) and 70–74 (*p* = 0.008) years was found. In 200 m, there was a decrease in sex difference in age groups 40–44 (*p* = 0.044) and 90–94 (*p* = 0.011) years, but sex difference increased significantly in age group 25–29 (*p* = 0.006) years.

## Discussion

This study investigated participation and performance trends in age group backstroke swimmers competing at FINA World Masters Championships over a 29-year period. It was hypothesized that (1) participation of world class age group backstroke swimmers would increase, (2) performance would improve for all age groups, and (3) men would be faster than women in all age groups. The main findings were, (1), participation in most age groups increased with exception of female participation in age groups 55–59 and 60–64 years in 50 m, (2), overall swimming performance in all age groups from 25–29 to 95–99 years improved over time for all distances, and, (3), in age groups 25–29 to 80–84 years men were faster than women (except age groups 85–89 to 95–99 years) over time and all distances.

### Increase in participation of swimmers in older age groups

A first important finding was an increase in the number of participants in different age groups, in particular in 100 and 200 m in age groups 45–49 to 70–74 and 80–84 years. However, there are few studies focusing on backstroke, an increase in participation in age groups older than 40 years was recently reported in a large cross-sectional study investigating master freestyle swimmers (Knechtle et al. 2016). Apart from swimming these findings were similar for marathon (Jokl et al. 2004; Lepers and Cattagni 2012) and ultra-marathon-running (Jampen et al. 2013; Rüst et al. 2013; Zingg et al. 2013), Ironman triathlon (Lepers et al. 2013; Stiefel et al. 2014), Deca Iron ultra-triathlon (Knechtle et al. 2012) and long-distance inline-skating (Teutsch et al. 2013).

The increase in participation of swimmers competing in 100 m and 200 m backstroke in age groups 45–49 to 70–74 and 80–84 years and the fact that there even are participants in age groups 85–89 to 100–104 years might be explained by different factors. In the last decades there has been an increase in commercialization of sport events in general, which is closely associated with mass media (https://www.ukessays.com/essays/media/effect-of-commercialization-on-sporting-events-media-essay.php). Across the years from 1990 to 2012, there has been an increase in life expectancy in the general Western population (www.who.int/mediacentre/news/releases/2014/world-health-statistics-2014/en/). However, life expectancy worldwide has also risen in the last 45 years and population size is expected to rise worldwide further in the next 35 years (www.prb.org/Publications/Datasheets/2014/2014-world-population-data-sheet/data-sheet.aspx). Since 1970, people in the general population are getting older (www.prb.org/Publications/Datasheets/2014/2014-world-population-data-sheet/data-sheet.aspx) due to advances in medicine for example the high medication use (Chen et al. 2001) and better prosthesis (Mascarenhas and MacDonald 2008). The findings of an increase in participation in different age groups in backstroke swimming over 100 m and 200 m distance in older age groups may reflect the trend that there is an increasing number of people older than 65 years in a good health condition in the general population and physically able to participate in different sport competitions ranging from swimming (Senefeld et al. 2016), marathon (Jokl et al. 2004) and ultra-marathon running (Jampen et al. 2013; Rüst et al. 2013; Zingg et al. 2013), Ironman triathlon (Lepers et al. 2013; Stiefel et al. 2013), Triple and Deca Iron ultra-triathlon (Knechtle et al. 2012) to long-distance inline skating (Teutsch et al. 2013). Health benefits, social factors, physical fitness, competition, enjoyment and personal challenge are some reasons for masters’ participation in sport events (Reaburn and Dascombe 2008) and could reflect the trend of high participation in older swimmers.

The knowledge of physical activity in an aging population is of high interest. King and King (2010) focused on the benefits of regular physical activity for older adults. They reported that major chronic diseases of aging, which are responsible for a high proportion of global mortality, are considerably influenced by regular physical activity. Another potential explanation for the growing numbers of participants aged 65–69, 70–74 and 80–84 years in the last 29 years might be the fact that athletes who are retired have more time at their disposal for training. Eibich (2015) found that retirees have an active lifestyle and practice physical exercise more frequently.

### Improvement of swimming performance

The second finding of the present study was that swimming performance improved in athletes in all age groups from 25–29 to 95–99 years for all distances. Apart from backstroke, similar findings were reported for all age groups from 25–29 to 85–89 and both sexes for all distances for athletes competing in freestyle swimming (Knechtle et al. 2016). Several studies found an improvement in performance in different age groups over 40 years in other sport disciplines for example in marathon running (Anthony et al. 2014; Jokl et al. 2004; Knechtle et al. 2012), ultra-marathon running (Zingg et al. 2013), Ironman triathlon (Lepers et al. 2013; Stiefel et al. 2014) and long-distance inline-skating (Teutsch et al. 2013).

These improvements in swimming performance of athletes in age groups from 25–29 to 95–99 years might be explained by several reasons. Swimmers might have been training for and participating in swimming competitions at younger ages for several years and continued (www.washingtonpost.com/blogs/early-lead/wp/2015/04/06/meet-the-100-year-old-japanese-swimmer-who-set-a-1500-meter-world-record/; http://swimswam.com/jaring-timmerman-oldest-masters-swimmer-passes-away-105/). Previous experiences could be a possible reason for their improvement in swimming performance over time in contrast to newcomers in younger age groups. Aspects of training should be considered as well. Since specific training programs exist for older athletes regarding physical characteristics and health conditions (www.coach.ca/files/Coaching_Master_Athletes_FINAL_EN.pdf) older athletes might have better opportunities to improve their performance. Robertson et al. (2009) described the importance of lap times and their effect on final times in swimming competitions.

Apart from specific training programs the knowledge of optimal nutrition for these age groups and its impact on better performance in older athletes in the last years might be important. Optimal nutrition recommendations exist for swimmers (Shaw et al. 2014), especially for older athletes concerning the intake of proteins, carbohydrates, selected vitamins and minerals and fluids and the knowledge of a specific diet for older athletes could be used to enhance their performance (Campbell and Geik 2004). A further potential explanation for the improvement of swimming performance in age groups 25–29 to 95–99 years is that apart from swimming, several master athletes may practice other endurance sport disciplines for example running and cycling when they prepare for an Ironman triathlon (Lepers et al. 2013; Stiefel et al. 2013, 2014).

### Sex differences in performance

A further interesting result was that men were faster than women in age groups from 25–29 to 80–84 years. Similar results were reported in swimming (Senefeld et al. 2016), in particular in master freestyle swimming where men were faster than women in age groups from 25 to 79 years (Knechtle et al. 2016). Although there are differences between sports, our findings that sex difference decreased in 50 m and 100 m in age groups 40–44 to 55–59 are in line with findings for Ironman triathlon. Lepers et al. (2013) reported that sex differences decreased in all age groups between 40–44 and 55–59 years in Ironman triathlon.

These findings in the present study might be explained by different factors. A potential explanation for faster race times in men in age groups from 25–29 to 80–84 years might be the fact that men have a higher skeletal muscle mass than women, especially in the upper body (Janssen et al. 2000). In comparison to women, muscle strength and muscle endurance are superior in men (Sugimoto et al. 2014). Puts et al. (2005) reported that women are frailer than men and frailty is associated to a greater extend with mortality in women than in men. Biomechanical factors such as stroke rate and stroke length should be considered. Kennedy et al. (1990) analyzed male and female Olympic swimmers and found that men were taller, had longer stroke lengths and higher stroke rates.

Sex differences in swimming performance and the fact that women in age groups 85–89 to 95–99 years were faster than men might be explained by the worldwide higher life expectancy in women than in men (www.prb.org/Publications/Datasheets/2014/2014-world-population-data-sheet/data-sheet.aspx). However, there are several biological factors contributing to the higher life expectancy in women in comparison to men, it is difficult to identify the relative importance of any one factor (Seifarth et al. 2012).

Although master athletes are committed, have high perceptions of ability and belonging as well as a high intrinsic motivation (Hodge et al. 2008), differences in motivation for sport participation exist between women and men (Kilpatrick et al. 2005). Kilpatrick et al. (2005) reported that women are more focused on body weight status, while men are highly motivated by performance and ego-related factors (*i.e.* strength, endurance, challenge, competition and social recognition) in comparison to women.

### Strengths and weaknesses

To our knowledge, the present study provides the first data on trends in participation and performance in age group master swimmers over a time-period of 29 years for backstroke swimming. Particular strengths of the present study are the large number of swimmers investigated, the long investigated time-period and the fact that even athletes competing very old age groups up to 100–104 years were considered. A further strength is that we did not select to the top swimmers but respected all swimmers ranked in their age group. This eliminates a selection bias when focusing only on the top athletes. A potential weakness of our study might be the fact that the cross-sectional design does not allow drawing conclusions on the individuals’ participation and performance in an age group over time. Despite this weakness, the present study may provide new insight into participation and performance trends of master swimmers for a particular swim style.

### Limitations and implications for future research

In the present study we did not consider other performance-related factors such as training (www.coach.ca/files/Coaching_Master_Athletes_FINAL_EN.pdf), motivation (Hodge et al. 2008; Kilpatrick et al. 2005; Reaburn and Dascombe 2008), previous experience (www.washingtonpost.com/blogs/early-lead/wp/2015/04/06/meet-the-100-year-old-japanese-swimmer-who-set-a-1500-meter-world-record/; http://swimswam.com/jaring-timmerman-oldest-masters-swimmer-passes-away-105/) or nutrition (Campbell and Geik 2004; Shaw et al. 2014). Since data on trends of participation and performance in different age groups over time are still missing, further studies should be performed to expand the knowledge on other strokes.

### Practical applications

The results that participation increased and performance improved in different age groups in backstroke swimming over time, especially in very old age groups, are interesting findings for both coaches and athletes. Not only do these results reinforce athletes to continue training for and participating in master competitions, but also should coaches continue promoting master athlete’s motivation for training for and participating in such competitions. Since the improvement in older and very old age groups might be the result of regular training over years, coaches should develop specific training plans for lifelong training in different age groups. Another possible challenge for coaches might be in the development of specific training programs focusing on men in age groups 85–89 to 95–99 years to help reduce differences in performance between women and men in these age groups.

## Conclusion

To summarize, in the master backstroke swimmers, participation increased in different age groups over time with the exception of women in age groups 55–59 and 60–64 years in 50 m, swimming performance improved in all age groups from 25–29 to 95–99 years over all distances, and men were faster than women in age groups from 25–29 to 80–84 years (except age groups 85–89 to 95–99 years) over time and all distances. Based upon this analysis, we assume a persistent trend of increased participation and improved performance in very old age group swimmers. Further investigations are required in order to analyse participation and performance trends of master athletes in backstroke swimming as well as in other swim strokes.
